# Multisensory emotion perception in congenitally, early, and late deaf CI users

**DOI:** 10.1371/journal.pone.0185821

**Published:** 2017-10-12

**Authors:** Ineke Fengler, Elena Nava, Agnes K. Villwock, Andreas Büchner, Thomas Lenarz, Brigitte Röder

**Affiliations:** 1 Biological Psychology and Neuropsychology, Institute for Psychology, Faculty of Psychology and Human Movement Science, University of Hamburg, Hamburg, Germany; 2 German Hearing Centre, Department of Otorhinolaryngology, Medical University of Hannover, Hannover, Germany; University of Montreal, CANADA

## Abstract

Emotions are commonly recognized by combining auditory and visual signals (i.e., vocal and facial expressions). Yet it is unknown whether the ability to link emotional signals across modalities depends on early experience with audio-visual stimuli. In the present study, we investigated the role of auditory experience at different stages of development for auditory, visual, and multisensory emotion recognition abilities in three groups of adolescent and adult cochlear implant (CI) users. CI users had a different deafness onset and were compared to three groups of age- and gender-matched hearing control participants. We hypothesized that congenitally deaf (CD) but not early deaf (ED) and late deaf (LD) CI users would show reduced multisensory interactions and a higher visual dominance in emotion perception than their hearing controls. The CD (n = 7), ED (deafness onset: <3 years of age; n = 7), and LD (deafness onset: >3 years; n = 13) CI users and the control participants performed an emotion recognition task with auditory, visual, and audio-visual emotionally congruent and incongruent nonsense speech stimuli. In different blocks, participants judged either the vocal (Voice task) or the facial expressions (Face task). In the Voice task, all three CI groups performed overall less efficiently than their respective controls and experienced higher interference from incongruent facial information. Furthermore, the ED CI users benefitted more than their controls from congruent faces and the CD CI users showed an analogous trend. In the Face task, recognition efficiency of the CI users and controls did not differ. Our results suggest that CI users acquire multisensory interactions to some degree, even after congenital deafness. When judging affective prosody they appear impaired and more strongly biased by concurrent facial information than typically hearing individuals. We speculate that limitations inherent to the CI contribute to these group differences.

## Introduction

The emotional signal is crossmodal in nature as emotions are conveyed for example by affective prosody and facial expressions [1,2].

In particular, redundant/congruent emotional information coming from faces and voices has been found to facilitate emotion recognition, while non-redundant/incongruent emotional information (e.g., a “happy” face and a “sad” voice) impairs judgments about the emotion expressed in one modality [1,3,4], even when response biases were controlled [5]. The finding that individuals cannot inhibit the processing of emotional input of a task-irrelevant modality has been taken as evidence for automatic interactions of crossmodal emotional signals (e.g., [4–6]).

In support of this notion, developmental studies have shown that by the age of 5–7 months, infants are able to detect common emotions across sensory modalities when presented with unfamiliar face-voice pairings [7–9]. This result is further corroborated by electrophysiological data collected in 7-months olds: The event-related potentials (ERPs) measured after presenting face-voice pairings differed for emotionally congruent and incongruent crossmodal stimuli [10]. These results suggest that multisensory interactions of emotional signals emerge early in development.

A recent theory on multisensory development, the multisensory perceptual narrowing (MPN) approach, assumes that multisensory functions are initially broadly tuned. Based on experience, they are differentiated resulting in an improvement of some multisensory functions and a loss of others [11]. In line with this idea, it has been suggested that multisensory processes depend on (multi)sensory input during sensitive periods [12,13]. As a consequence, impaired multisensory processing is expected after a transient phase of sensory deprivation from birth. Evidence for this assumption comes from studies including individuals with congenital sensory deprivation after sight restoration (e.g., [14]) or congenitally deaf cochlear implant (CI) users (e.g., [15]).

Cochlear implantation is performed at different stages of development and in individuals with both congenital and acquired deafness. In the latter individuals, deafness onset may be early (“prelingual”; before the age of 3 years) or late (“postlingual”; after the age of 3 years) [16,17]. Thus, studies with CI users specifically allow addressing the impact of auditory and related multisensory experience at different stages of development on multisensory processing.

To date, most research with CI users has focused on speech perception, which is—just as emotion perception—an inherently multisensory task [18,19]. It has been shown that CI users commonly have limited auditory-only speech recognition abilities and tend to rely more on the visual modality, which has been inferred particularly from responses to audio-visual incongruent speech (e.g., [15,20,21]). However, this finding appears to be modulated by several factors. First, speech recognition negatively relates to both age at deafness onset and age at implantation [16,17,22]. Specifically, auditory input within the first 3–4 years of life seems to be crucial for the typical development of speech perception, in line with the theory of early sensitive periods for uni- and multisensory development [12,15,22–24]. Second, so-called “proficient” CI users (i.e., individuals with at least 70% correct responses in standard auditory-only speech recognition measures) appear to be less biased by concurrent incongruent visual input as compared to their non-proficient counterparts [20,25]. In sum, it appears that the lack of auditory experience in early life results in worse auditory speech recognition and a higher visual dominance in multisensory speech perception as compared to later auditory deprivation.

The perception of paralinguistic information and particularly emotion perception has rarely been investigated in CI users. The existing research has mostly focused on unisensory emotion perception. These studies have suggested impairments in recognizing affective prosody in CI users with either early or late deafness onset [26–28]. By contrast, results for affective facial expression recognition are more inconsistent: Some investigators have found typical performance, whereas others have found lower performance in early deaf (ED) CI users as compared to controls [29–31]. Thus, these studies would suggest that multisensory emotion processing is dominated by visual cues in CI users. However, it is not known whether congenitally deaf (CD) CI users show any interaction of auditory and visual emotion cues and if yes whether the degree depends on the age at cochlear implantation.

The only studies that—to our knowledge—have investigated multisensory emotion perception in CI users yet were conducted in early deaf (ED) children and adolescents [32,33]. Most & Aviner [32] assessed auditory, visual, and congruent audio-visual emotion recognition in participants aged between 10 and 17 years, implanted either before or after the age of 6 years. Participants had to judge the emotion expressed in 36 video recordings of a professional actor speaking out the same neutral sentence (“I am going out now and I’ll be back later”) with different affective prosody and facial expressions (anger, fear, sadness, happiness, disgust, and surprise). In the auditory condition, the audio track was presented alongside with a black screen; in the visual condition, the video track was presented without sound; in the audio-visual condition, the video and audio recordings were presented concurrently. Results showed that in the auditory condition, the typically hearing control group performed better than both CI groups (implanted early and late, respectively); the CI groups did not differ in performance. All participants achieved higher performance in the visual as compared to the auditory condition. Furthermore, all participants scored higher in the audio-visual as compared to the auditory condition. In contrast, only the control group showed enhanced recognition performance in the audio-visual relative to the visual condition.

Using a similar study design, Most & Michaelis [33] showed that ED children with CI (early implanted), aged between 4 and 6 years, performed worse relative to controls in auditory and audio-visual emotion recognition. CI users and controls did not differ with respect to visual emotion recognition. All groups performed better in the audio-visual condition as compared to the auditory and the visual condition, respectively. The unimodal conditions did not differ in either group.

Together, the results of these two studies suggest that ED children and adolescents with CIs benefit from congruent audio-visual as compared to auditory emotional information, although the level of audio-visual emotion recognition might be decreased relative to controls. Furthermore, these individuals seem to be impaired in affective prosodic recognition, but possess typical facial expression recognition abilities. Finally, the two studies suggest that the benefit from congruent vocal cues for facial expression recognition might change with age or with CI experience, as a gain from the additional vocal information was found in children but not in adolescents with CIs. Importantly, all previous studies distinguished only between ED and late deaf (LD) CI users. However, research in individuals with congenital dense cataract, who recovered sight at different stages in early development, has demonstrated the crucial role of visual experience during the first months of life for the functional development of (multi)sensory processing [14, 34]. In light of this evidence, it might be assumed that CD CI users recover less than CI users with early or late deafness onset.

These two open questions were addressed with the present study: We investigated the performance of adolescent and adult CD, ED, and LD CI users, age- and gender-matched with three groups of typically hearing control participants, in an emotion recognition task with auditory, visual, and audio-visual emotionally congruent as well as emotionally incongruent non-sense speech stimuli. Incongruent crossmodal stimuli were included as well since the automaticity of multisensory interactions can only be assessed with this condition (see [5], analogously to studies on audio-visual speech perception). In different blocks, participants judged the emotional expression of either the voices (i.e., affective prosodic recognition) or the faces (i.e., affective facial expression recognition).

With reference to results from multisensory speech recognition in CI users and based on the concept of sensitive periods for uni- and multisensory functions, we hypothesized that CD, but not ED and LD, CI users would show reduced multisensory interactions and a higher visual dominance in emotion perception as compared to their control participants.

## Materials and methods

### Participants

Seven CD, 7 ED (deafness onset <3 years), and 15 LD (deafness onset >3 years) adolescent and adult CI users as well as age- and gender matched typically hearing controls (7 for the CD CI users, 7 for the ED CI users, and 13 for the LD CI users) took part in this study.

The CI users were recruited from the German Hearing Centre of the Medical University of Hannover as well as through self-help groups and personal contacts. The control participants were recruited from the University of Hamburg and the local community. Adult participants gave written informed consent prior to participation and received either course credits or a monetary compensation. Minor participants gave oral informed consent and written informed consent was obtained from their parents or guardians. Minors received a small present for their participation. In addition, costs for travelling and/or accommodation were covered for the CI users.

The study was approved by the ethical committee of the DGPs (Deutsche Gesellschaft für Psychologie, no. BR 07_2011) and by the ethical committee of the Medical University Hannover (no. 1191–2011). The principles expressed in the Declaration of Helsinki (2013) were followed.

The data of two CI users and one control participant were discarded from the final sample because their performance deviated more than 2 SD from their group’s mean. The final sample of CI users thus included 7 CD CI users (3 females; mean age: 24 years, age range: 15–44 years; mean age at implantation: 12 years, range 1–42 years), 7 ED CI users (5 females; mean age: 25 years, age range: 19–43 years; mean age at deafness onset: 2 years, range: 0.5–3 years; mean age at implantation: 9 years, range: 2–33 years), and 13 LD CI users (6 females; mean age: 48 years, age range: 28–60 years; mean age at deafness onset: 40 years, range: 18–50 years; mean age at implantation: 41 years, range: 23–50 years). The majority of the CI users in each group were implanted bilaterally (see Table 1). The final sample of typically hearing control participants included 7 controls for the CD CI users (3 females; mean age: 24 years, age range: 14–47 years), 7 controls for the ED CI users (5 females; mean age: 26 years, age range: 19–45 years), and 12 controls for the LD CI users (6 females; mean age: 47 years; age range: 28–63 years). All participants reported normal or corrected-to-normal vision, and the control participants reported normal hearing.

**Table 1:** Characteristics of CI users included in the study.

ID	Gender	Age (years)	Age at deafness onset	Age at implantation (first CI, years)	Duration of deafness (years)	CI experience (years)	Implant side	Type of the CI(s)
**CD 1**	m	20	0	2	2	18	right	Cochlear
**CD 2**	f	24	0	10	10	14	bilateral	Advanced Bionics
**CD 3**	m	19	0	2	2	17	right	Advanced Bionics
**CD 4**	m	15	0	1	1	14	bilateral	Cochlear
**CD 5**	f	26	0	16	16	10	bilateral	Cochlear
**CD 6**	f	44	0	42	42	2	left	Cochlear
**CD 7**	m	23	0	8	8	15	bilateral	Cochlear
**ED 1**	f	22	3	4	1	18	bilateral	Cochlear
**ED 2**	f	19	2	3	1	16	bilateral	Advanced Bionics
**ED 3**	m	26	0.5	6	5.5	20	right	Cochlear
**ED 4**	f	23	1.5	2	0.5	21	right	Cochlear
**ED 5**	f	24	3	10	7	14	bilateral	Cochlear
**ED 6**	m	43	1.5	33	31.5	10	bilateral	Advanced Bionics
**ED 7**	f	19	3	3	0.5	16	bilateral	Cochlear
**LD 1**	f	59	50	50	1	9	bilateral	Advanced Bionics
**LD 2**	m	58	49	50	1	8	bilateral	Cochlear
**LD 3**	m	60	50	50	1	10	right	Advanced Bionics
**LD 4**	m	53	41	42	1	11	bilateral	Advanced Bionics
**LD 5**	m	36	30	31	1	5	left	Cochlear
**LD 6**	f	56	49	50	1	6	bilateral	Advanced Bionics
**LD 7**	f	53	45	47	2	6	bilateral	Cochlear
**LD 8**	m	52	43	44	1	8	bilateral	MED-El
**LD 9**	m	51	45	46	1	5	bilateral	MED-El
**LD 10**	f	41	32	33	1	8	bilateral	Cochlear
**LD 11**	f	28	18	23	5	5	right	Cochlear
**LD 12**	m	45	30	32	2	13	bilateral	Advanced Bionics
**LD 13**	f	38	32	33	1	5	bilateral	Cochlear

Displayed are individual values of CI participants from the three groups. CD = congenitally deaf; ED = early deaf; LD = late deaf.

### Stimuli and procedure

The task was adapted from [5] where details of the stimulus generation and evaluation are described. Auditory stimuli consisted of short sound tracks of voices speaking out bisyllabic German pseudowords (“lolo”, “tete”, or “gigi”) at a sound level varying between 65 and 72 dB, presented via two loudspeakers located at either side of a computer screen (width = 36 cm). On the screen, the visual stimuli, consisting of short video clips showing faces mouthing the same bisyllabic pseudowords, were presented at 60 cm viewing distance (width = 4° of visual angle; height = 9° of visual angle). The outer facial features (i.e., hair and ears) were covered to maximize attention to the inner facial features (i.e., eyes and mouth). The affective prosody of the voices and the facial expressions were happy, sad, angry, or neutral. Participants were provided with written instructions and a keyboard for response registration. They were instructed to respond as quickly and as accurately as possible using their dominant hand throughout the experiment. Stimulus presentation and response recording was effected via the Presentation program by Neurobehavioral Systems.

Unimodal auditory and visual stimuli were voices and faces presented alone, respectively. Audio-visual congruent trials presented face-voice pairings with voice and face displaying the same emotion (e.g., “happy” voice and face) and audio-visual incongruent trials presented face-voice pairings with voice and face displaying a different emotion (e.g., “happy” voice and “angry” face). For both congruent and incongruent audio-visual trials the audio track and the video stream originated from independent recordings. This allowed compensating for possible minimal temporal misalignments when independent audio and visual streams were combined ([5], for details).

Each trial began with a 500 ms audio-visual warning signal (a grey circle of 2° of visual angle, combined with multi-talker babble noise) to orient the participants’ attention to the stimuli. After a variable inter-stimulus interval (ISI; 600–700 ms, uniform distribution), participants were presented with a voice alone (i.e., an audio track), a face alone (i.e., a video stream), or a face-voice pairing (i.e., video and audio stream). In half of the experimental blocks, the participants had to recognize the emotion and rate the intensity of the emotional expression of the voice while ignoring the visual input (Voice task). In the other half of the blocks, they had to recognize the emotion and rate the intensity of the emotional expression of the face while ignoring the auditory input (Face task). While both tasks enclosed audio-visual congruent and audio-visual incongruent trials, the Voice task additionally enclosed unimodal auditory trials and the Face task additionally comprised unimodal visual trials.

Each stimulus was presented twice, with an ISI of 3 s. Following the first presentation, participants judged the displayed emotion by pressing one of four adjacent marked buttons on the keyboard. After the second presentation, participants rated the intensity of the emotional expression. To this end, a rating scale was presented on the screen (50 ms after stimulus offset) that ranged from 1 (low) to 5 (high) and the participants typed in one of the corresponding numbers on the keyboard.

Up to 10 practice trials were run to familiarize the participants with the task. The number of practice trials was chosen to guarantee a sufficient familiarity with the task and stimuli across all groups. The experiment took on average 40 min to complete.

### Data analysis

For each participant, we calculated accuracy rates (in percent), reaction times (RTs), and emotion intensity ratings (possible range: 1–5), separately for condition (i.e., unimodal, audio-visual congruent, audio-visual incongruent) and task (i.e., Voice task, Face task). To account for possible speed-accuracy trade-offs, we derived inverse efficiency scores (IES) by dividing mean RTs by the proportion of correct trials [35]. Separate analyses on accuracy rates and RTs are provided in the Supporting information (S1 and S2 Text).

IES and intensity ratings were analyzed with a 2 (Group: CI users, controls) × 3 (Condition: unimodal, audio-visual congruent, audio-visual incongruent) analysis of variance (ANOVA) for each CI group and its respective control group, separately for each task (i.e., Voice task, Face task). Violation of sphericity for the repeated measurement factor Condition was accounted for by the Huynh-Feldt correction. In order to demonstrate that we were able to replicate the effects of crossmodal stimulation in the Voice and the Face task as reported earlier [5], we additionally ran a repeated measures ANOVA with Condition as factor for the complete group of controls (n = 26) for each task.

Post hoc tests on main effects were computed by Welch Two Sample *t*-tests (for group comparisons) and Holm-Bonferroni corrected paired *t*-tests (for comparisons of conditions), respectively. Interaction effects (Group x Condition) were further analyzed by testing group differences in each condition and condition differences in each group. In addition, we compared congruency and incongruency effects (i.e., difference between the congruent and incongruent as compared to the unimodal condition, respectively) between the CI users and their respective controls. This procedure permitted a direct comparison between CI users and controls with respect to the modulatory effect of stimulation condition (cross-modal [in]congruent vs. unimodal) on emotion perception.

## Results

### IES: Voice task

*All control participants (n = 26)*: The ANOVA showed that the repeated measures factor Condition was significant (*F*(2, 50) = 14.63, *p* < .001). Post hoc tests revealed that the controls were significantly more efficient in the congruent condition as compared to the unimodal condition (*t*(25) = -3.69, *p* < .01; congruency effect) and the incongruent condition (*t*(25) = -6.27, *p* < .001). The unimodal and incongruent conditions did not significantly differ (*t*(25) = -1.37, *p* = .18; no incongruency effect).

*CD CI users and controls for CD CI users*: The ANOVA displayed a main effect of Group (*F*(1, 12) = 24.21, *p* < .001), indicating that the CD CI users performed overall less efficiently than their matched controls (CD CI users: mean = 5227.49 ms, SD = 3276.44 ms; controls: mean = 2223.52 ms, SD = 555.22 ms). There was a significant main effect of Condition as well (*F*(2, 24) = 11.18, *p* = .001) and a Group x Condition interaction (*F*(2, 24) = 8.52, *p* < .01), suggesting that the effect of Condition on IES differed between the groups.

**Fig 1:**
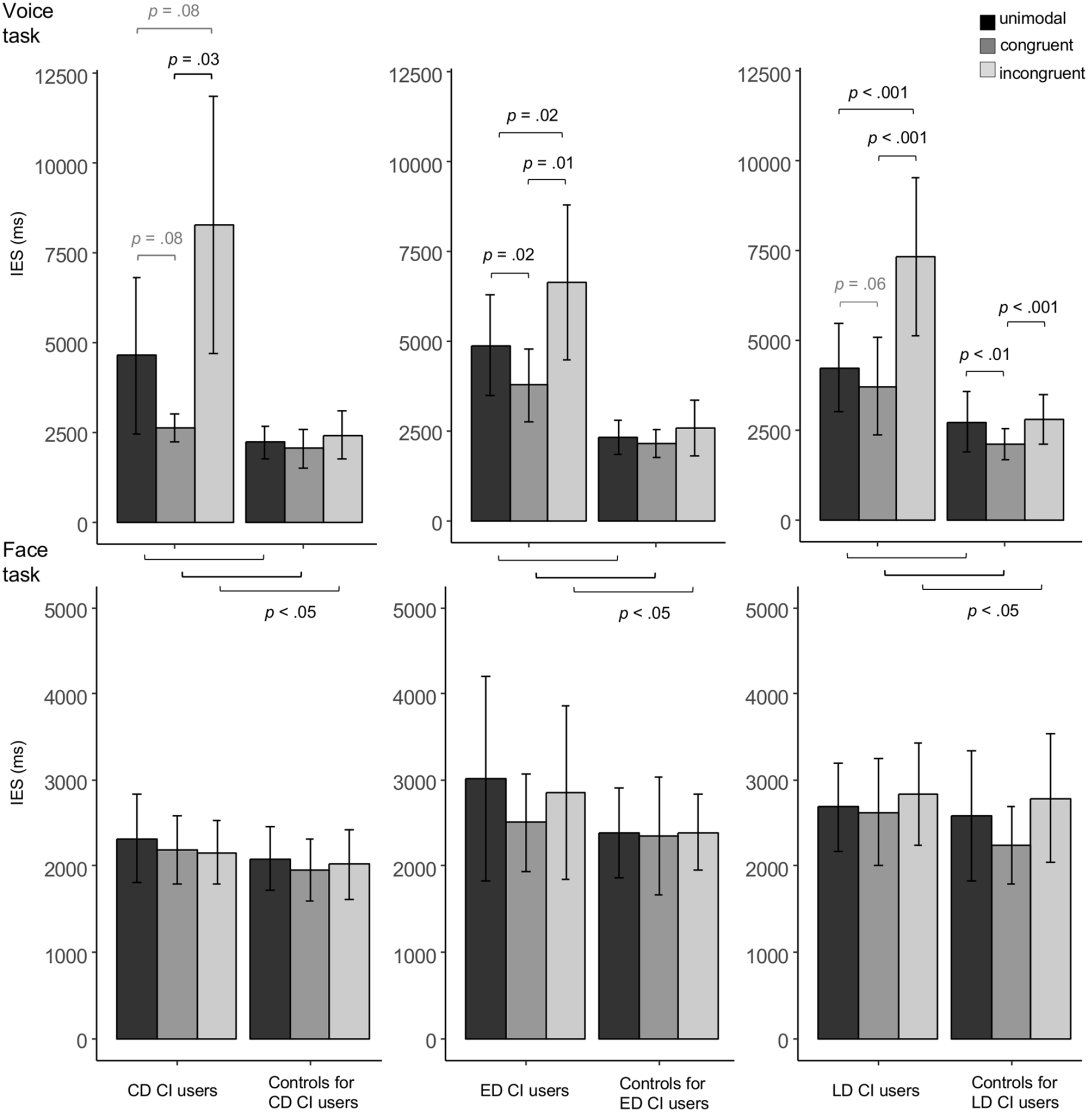
IES in the voice and the face task. Inverse efficiency scores (IES, ms) in the CD (n = 7), ED (n = 7), and LD (n = 13) CI users and their respective controls, separately for task (Voice task, Face task) and condition (unimodal, congruent, incongruent). Error bars denote standard deviations. Please note the different scales in the Voice and the Face task. P-values indicate (marginally) significant group differences per condition and condition differences per group.

Post hoc group comparisons for each condition showed that the CD CI users performed less efficiently than their controls in all three conditions (all *t*-tests *p* < .05; see Fig 1). Repeated measures ANOVA on the effect of Condition showed that the conditions significantly differed in the CD CI users (*F*(2, 12) = 10.11, *p* < .01), but not in the controls (*F*(2, 12) = 1.51, *p* = .26). As illustrated in Fig 1, the CD CI users were significantly more efficient in the congruent relative to the incongruent condition (*t*(6) = -3.70, *p* = .03), and marginally significantly more efficient in the congruent compared to the unimodal condition (*t*(6) = -2.59, *p* = .08). Furthermore, they performed marginally significantly more efficiently in the unimodal compared to the incongruent condition (*t*(6) = -2.61, *p* = .08).

**Fig 2:**
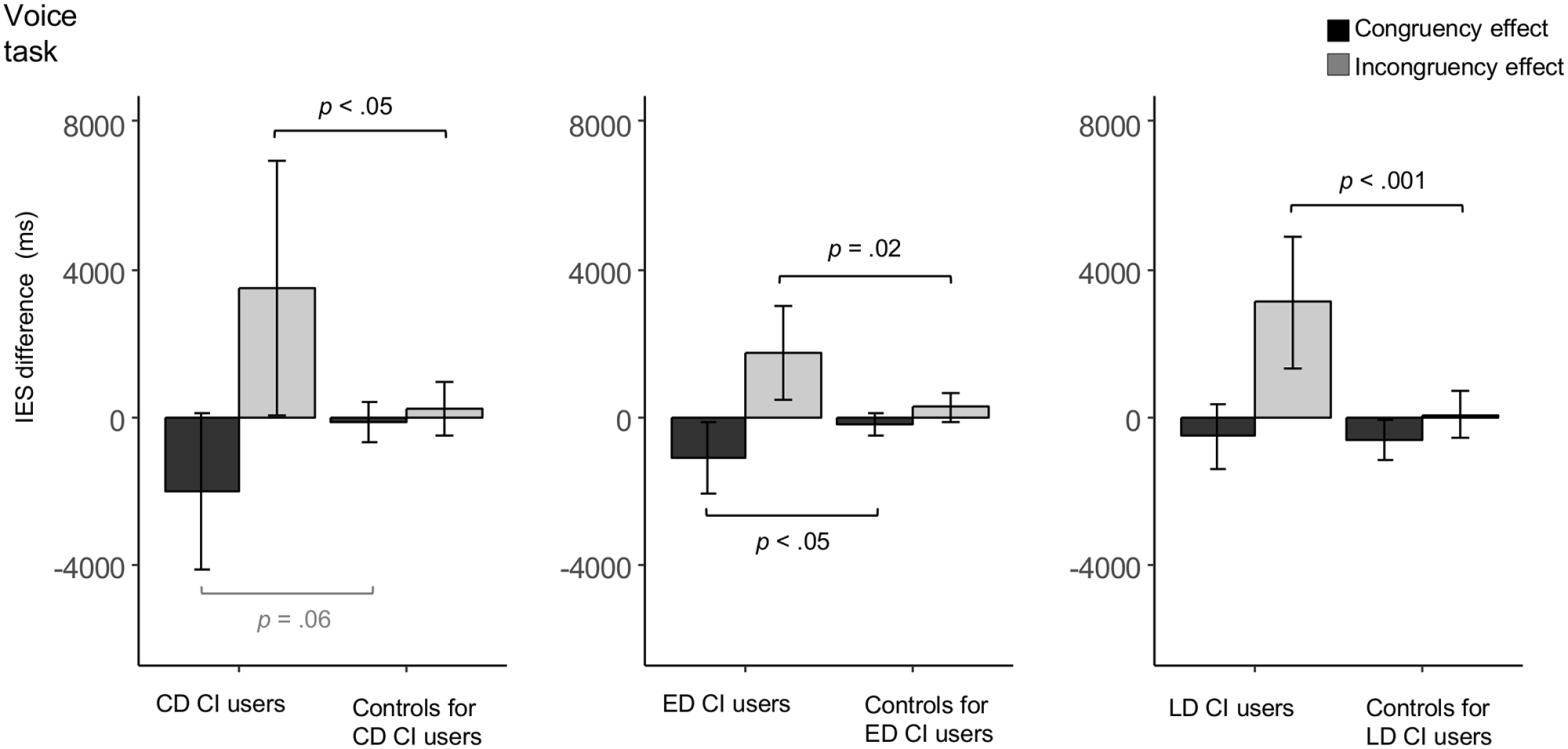
IES (In)congruency effects (voice task). Congruency and incongruency effects in inverse efficiency scores (IES, ms) in the CD (n = 7), ED (n = 7), and LD (n = 13) CI users and their respective controls in the Voice task. Error bars denote standard deviations. (Marginally) significant group differences are indicated accordingly.

Comparisons of the congruency and incongruency effects (i.e., IES differences between the congruent/incongruent and the unimodal condition) affirmed group differences concerning the effect of Condition on IES: The CD CI users had a marginally significantly larger congruency effect (*t*(6) = -2.25, *p* = .06) and a significantly larger incongruency effect (*t*(6) = 2.44, *p* < .05) as compared to their controls (see Fig 2).

Additional correlational analyses showed that the (in)congruency effects were independent of age at implantation in the CD CI users. Neither the congruency effect and age at implantation correlated significantly (*t*(5) = -0.93, *p* = .40), nor did the incongruency effect and age at implantation (*t*(5) = 1.47, *p* = .20).

*ED CI users and controls for ED CI users*: The ANOVA showed a main effect of Group (*F*(1, 12) = 23.47, *p* < .001), indicating overall higher IES in the ED CI users than in their controls (ED CI users: mean = 5086.94 ms, SD = 1943.80 ms; controls: mean = 2342.93 ms, SD = 576.19 ms). The main effect of Condition was significant too (*F*(2, 24) = 19.38, *p* < .001) as well as a Group x Condition interaction (*F*(2, 24) = 10.33, *p* < .01), suggesting that the effect of Condition on IES differed between the groups.

In post hoc comparisons, the ED CI users were found to perform significantly less efficiently than their controls in all three conditions (all *t*-tests *p* < .05; see Fig 1). Repeated measures ANOVA showed that the effect of Condition was significant in both the ED CI users (*F*(2, 12) = 15.68, *p* = .001) and their controls (*F*(2, 12) = 4.70, *p* = .03). As illustrated in Fig 1, the ED CI users performed significantly more efficiently in the congruent as compared to both the unimodal condition (*t*(6) = -3.05, *p* = .02) and the incongruent condition (*t*(6) = -4.37, *p* = .01). Furthermore, they performed significantly more efficiently in the unimodal than in the incongruent condition (*t*(6) = -3.61, *p* = .02). In the controls, none of the post hoc comparisons yielded significant condition differences.

Comparisons of the congruency and incongruency effects (i.e., IES differences between the congruent/incongruent and the unimodal condition) confirmed that the effect of Condition on IES differed between the ED CI users and their controls: The ED CI users displayed significantly larger congruency (*t*(7) = -2.43, *p* < .05) and incongruency (*t*(7) = 2.94, *p* = .02) effects as compared to their controls (see Fig 2).

*LD CI users and controls for LD CI users*: The ANOVA displayed a main effect of Group (*F*(1, 23) = 35.14, *p* < .001), indicating that the LD CI users had overall higher IES than their controls (LD CI users: mean = 5089.85 ms, SD = 2288.70 ms; controls: mean = 2534.91 ms, SD = 721.60 ms). The main effect of Condition (*F*(2, 46) = 44.64, *p* < .001) and the interaction of the Group x Condition were significant as well (*F*(2, 46) = 25.19, *p* < .001), suggesting that the effect of Condition on IES differed between the groups.

In post hoc comparisons, the LD CI users were shown to perform less efficiently than their controls in all three conditions (all *t*-tests *p* < .05; see Fig 1). Repeated measures ANOVA indicated that the effect of Condition was significant in both the LD CI users (*F*(2, 24) = 38.91, *p* < .001) and their controls (*F*(2, 22) = 11.55, *p* < .001). As illustrated in Fig 1, the LD CI users’ performance was marginally significantly more efficient in the congruent as compared to the unimodal condition (*t*(12) = -2.13, *p* = .06) and significantly more efficient in both the unimodal and the congruent as compared to the incongruent condition, respectively (*t*(12) = -6.34, *p* < .001; *t*(12) = -6.71, *p* < .001). The controls had significantly lower IES in the congruent as compared to the unimodal and the incongruent condition, respectively (*t*(11) = -3.88, *p* < .01; *t*(11) = -5.78, *p* < .001), whereas the unimodal and incongruent conditions did not significantly differ (*t*(11) = -0.36, *p* = .72).

Comparisons of the congruency and incongruency effects (i.e., IES differences between the congruent/incongruent and the unimodal condition) demonstrated that the LD CI users and their controls differed with regard to the effect of Condition on IES (see Fig 2): The LD CI users displayed a significantly larger incongruency effect as compared to their controls (*t*(15) = 5.80, *p* < .001). The congruency effect did not significantly differ between the LD CI and their controls (*t*(20) = 0.31, *p* = .76).

### IES: Face task

*All control participants (n = 26)*: The ANOVA showed that the repeated measures factor Condition was significant (*F*(2, 50) = 5.58, *p* < .01). Post hoc comparisons revealed that the controls were marginally significantly more efficient in the congruent condition as compared to the unimodal condition (*t*(25) = -2.25, *p* = .07; marginally significant congruency effect). They were significantly more efficient in the congruent as compared to the incongruent condition (*t*(25) = -2.89, *p* = .02). The unimodal and incongruent conditions did not significantly differ (*t*(25) = -1.08, *p* = .29; no incongruency effect).

**Fig 3:**
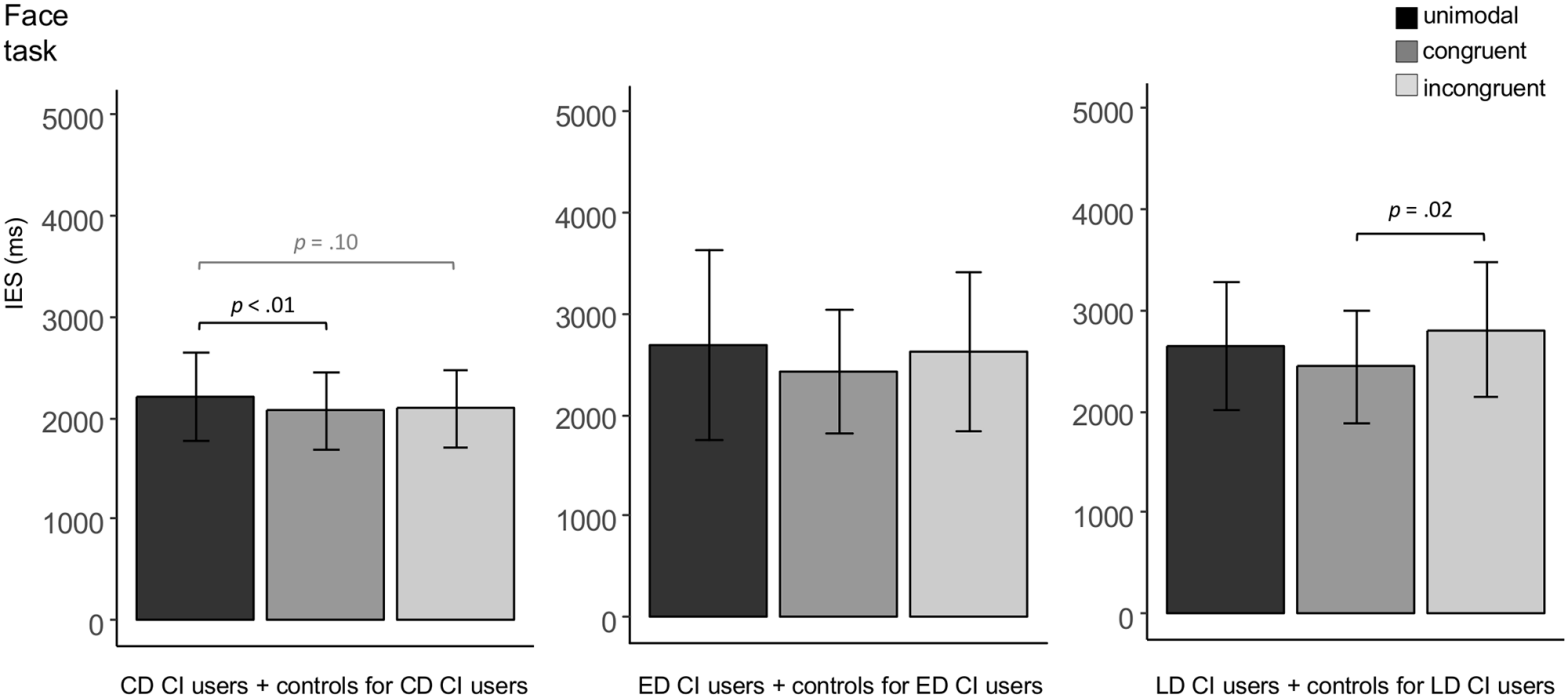
IES condition differences (face task). Inverse efficiency scores (IES, ms) in each condition (unimodal, congruent, incongruent) of the Face task in the CD CI users and their controls (n = 14), ED CI users and their controls (n = 14), and LD CI users and their controls (n = 25). Error bars denote standard deviations. (Marginally) significant condition differences are indicated accordingly.

*CD CI users and controls for CD CI users*: The ANOVA showed a significant main effect of Condition (*F*(2, 24) = 4.78, *p* = .03). Post hoc comparisons revealed that the CD CI users and their controls responded significantly more efficiently in the congruent as compared to the unimodal condition (*t*(13) = 4.45, *p* < .01). There was a trend towards more efficient recognition performance in the incongruent as compared to the unimodal condition as well (*t*(13) = 2.18, *p* = .10). The congruent and incongruent conditions did not significantly differ (*t*(13) = -0.19, *p* = .85; see Fig 3).

*ED CI users and controls for ED CI users*: The ANOVA did not reveal any significant effect.

*LD CI users and controls for LD CI users*: The ANOVA displayed a main effect of Condition (*F*(2, 46) = 5.21, *p* = .01). Post hoc comparisons showed that both groups responded significantly more efficiently in the congruent as compared to the incongruent condition (*t*(24) = -2.97, *p* = .02). Neither the congruent nor the incongruent condition significantly differed from the unimodal condition, respectively (unimodal vs. congruent: *t*(24) = 1.74, *p* = .19, unimodal vs. incongruent: *t*(24) = -1.61, *p* = .19; see Fig 3).

### Intensity rating: Voice task

*All control participants (n = 26)*: The ANOVA showed that the repeated measures factor Condition was significant (*F*(2, 50) = 8.63, *p* < .001). Post hoc comparisons revealed that the controls rated the emotional intensity as significantly higher in the congruent condition as compared to the unimodal condition (*t*(25) = 2.39, *p* < .05; congruency effect) and the incongruent condition (*t*(25) = 3.83, *p* < .01). Furthermore, their emotional intensity ratings were marginally significantly higher in the unimodal as compared to the incongruent condition (*t*(25) = 1.87, *p* = .07; marginally significant incongruency effect).

**Fig 4:**
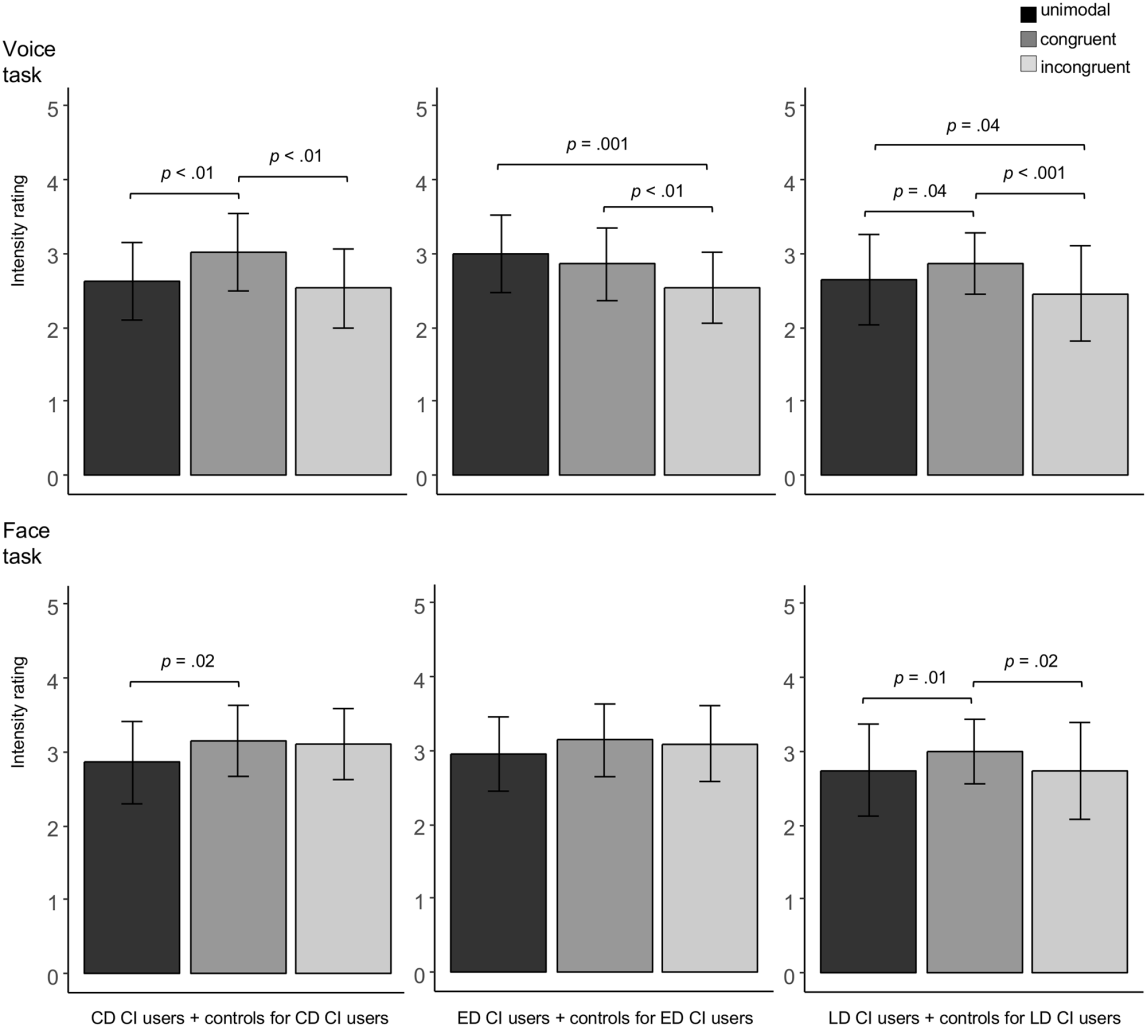
Perceived emotion intensity in the voice and the face task. Emotion intensity ratings (1 = *low*, 5 = *high*) in the CD CI users and their controls (n = 14), ED CI users and their controls (n = 14), and LD CI users and their controls (n = 25), separately for task (Voice task, Face task) and condition (unimodal, congruent, incongruent). Error bars denote standard deviations. (Marginally) significant condition differences are indicated accordingly.

*CD CI users and controls for CD CI users*: The ANOVA displayed a main effect of Condition (*F*(2, 24) = 14.28, *p* = .001). Post hoc comparisons revealed that the CD CI users and their controls rated the congruent stimuli as more intense than both the unimodal stimuli (*t*(13) = -4.58, *p* < .01) and the incongruent stimuli (t(13) = 3.88, *p* < .01). Perceived emotion intensity of the unimodal and incongruent stimuli did not significantly differ (*t*(13) = 1.10, *p* = .29; see Fig 4).

*ED CI users and controls for ED CI users*: The ANOVA displayed a main effect of Condition (*F*(2, 24) = 12.09, *p* < .001). Post hoc comparisons revealed that the ED CI users and their controls rated as more intense both the unimodal stimuli (*t*(13) = 5.14, *p* = .001) and the congruent stimuli (*t*(13) = 3.99, *p* < .01) as compared to the incongruent stimuli. No significant difference emerged between the congruent and the unimodal condition (*t*(13) = -1.33, *p* = .21; see Fig 4).

*LD CI users and controls for LD CI users*: The ANOVA displayed a main effect of Condition (*F*(2, 46) = 12.27, *p* < .001). Post hoc comparisons revealed higher emotion intensity ratings in the congruent condition as compared to both the unimodal condition (*t*(24) = -2.51, *p* = .04) and the incongruent condition (*t*(24) = 4.56, *p* < .001). Additionally, the LD CI users and their controls displayed significant higher intensity ratings in the unimodal condition as compared to the incongruent condition (*t*(24) = 2.46, *p* = .04; see Fig 4).

### Intensity rating: Face task

*All control participants (n = 26)*: The ANOVA showed that the repeated measures factor Condition was significant (*F*(2, 50) = 3.99, *p* = .02). Post hoc comparisons revealed that the controls rated the emotional intensity as higher in the congruent condition as compared to the unimodal condition (*t*(25) = 3.09, *p* = .01; congruency effect). The incongruent condition differed from neither the congruent condition (*t*(25) = 1.37, *p* = .18), nor the unimodal condition (*t*(25) = 1.34, *p* = .19; no incongruency effect).

*CD CI users and controls for CD CI users*: The ANOVA showed a significant main effect of Condition (*F*(2, 24) = 4.92, *p* = .02). Post hoc comparisons revealed that the CD CI users and their controls rated the congruent condition as significantly higher in intensity relative to the unimodal condition (*t*(13) = -3.31, *p* = .02), and the unimodal condition as marginally significantly higher in intensity than the incongruent condition (*t*(13) = -2.46, *p* = .06). The congruent and incongruent conditions did not significantly differ (*t*(13) = 0.37, *p* = .72; see Fig 4).

*ED CI users and controls for ED CI users*: The ANOVA did not reveal any significant effect.

*LD CI users and controls for LD CI users*: The ANOVA showed a significant main effect of Condition (*F*(2, 46) = 5.95, *p* < .01). Post hoc comparisons showed that the LD CI users and their controls rated the congruent condition as significantly more intense compared to both the unimodal condition (*t*(24) = -3.22, *p* = .01) and the incongruent condition (*t*(24) = 2.77, *p* = .02). The unimodal and incongruent conditions did not differ (*t*(24) = 0.00, *p* = .99; see Fig 4).

## Discussion

In the present study, we investigated the role of auditory experience at different stages of development for multisensory emotion perception in congenitally, early, and late deaf (CD, ED, and LD) adolescent and adult CI users.

The three groups of CI users, age- and gender-matched to three groups of typically hearing control participants, performed an emotion recognition task with unimodal auditory and visual as well as audio-visual emotionally congruent and incongruent speech stimuli. In different tasks, participants judged the emotional expression of either the voices (i.e., affective prosodic recognition) or the faces (i.e., affective facial expression recognition).

Consistently across tasks and dependent variables (inverse efficiency scores and intensity ratings) we found reliable congruency effects in the group of control participants. By contrast, incongruency effects were not reliably obtained.

Differences between the CI users and their matched control groups were found in the Voice task, pertaining to the efficiency of recognition (as assessed by inverse efficiency scores). Affective facial expression recognition efficiency as well as perceived vocal and facial emotion intensity did not differ between CI users and controls.

The CI users displayed generally impaired affective prosodic recognition efficiency as compared to their controls, irrespectively of age at deafness onset. This finding is in line with and complements previous studies showing that ED and LD CI users perform worse than controls in affective prosodic recognition (e.g., [26–28,32]). A possible reason for these results might be that hearing with a CI generally precludes a typical perception of affective prosody, probably due to the CI’s limitations in transferring low-frequency cues and pitch information [36–39]. In support of this idea, there is evidence that CI users with bimodal hearing support (i.e., one CI and a contralateral hearing aid) outperform uni- or bilaterally implanted CI users in affective prosodic recognition, suggesting that recognition of speech prosody might be limited by the CI *per se* [40–42].

Furthermore, all CI users experienced a higher interference from incongruent facial expressions on affective prosodic recognition efficiency than their controls. Finally, the CD and ED CI users benefitted (marginally) significantly more than their controls from congruent facial information to recognize affective prosody.

On the one hand, these results show that independently of age at deafness onset, CI users more strongly rely on facial expressions than hearing controls when presented with crossmodal emotional stimuli. In other words, multisensory emotion perception in CI users appears to be dominated by the visual modality, irrespectively of early experience with auditory and audio-visual stimuli. This finding contradicts our hypothesis that particularly CD CI users display a higher visual dominance relative to controls in multisensory emotion perception. Rather than being a consequence of the lack of auditory and/or audio-visual experience from birth, visually dominant emotion processing in CI users might more likely be due to the impoverished auditory signal provided by the CI.

Consistent with this idea, it has been theorized that humans process crossmodal information in a statistically optimal fashion. According to the maximum likelihood estimation (MLE) approach, the information from each sensory channel is weighted according to the signal’s reliability [43,44]. Indeed, studies on healthy individuals have demonstrated that decreasing the reliability of one sensory channel (for example by adding noise) reduces the weight of its information for the final percept (e.g., [45,46]). In CI users, the impaired affective prosodic recognition abilities found in several studies–including ours—indicate that vocal emotional signals are likely less reliable for these individuals than facial emotional signals. It can thus be argued that CI users allocate more weight to the visual channel than hearing individuals when presented with crossmodal emotional input, which may result in visually dominant percepts. Importantly, despite their stronger visual weight, CI users did not ignore the vocal signal when asked to recognize affective prosody in the presence of interfering facial information. All CI users achieved affective prosodic recognition accuracy levels significantly above zero in this condition (see S1 Text), indicating that they indeed considered the auditory input.

In contrast, the possibility that enhanced affective facial expression recognition skills of CI users underlie their visually dominant emotion perception appears unlikely considering that we did not observe any differences in recognition efficiency between the CI users and their matched controls in the Face task. Moreover, the accuracy and RT data from the Face task even suggest lower affective facial expression recognition accuracy in the CD CI users as compared to their controls (with no difference in RT) as well as longer RT in the LD CI users as compared to their controls (with no difference in accuracy; see displayed in S1 and S2 Figs). These results are in accordance with and add to previous results showing that CI users do not perform better than hearing controls in terms of affective facial expression recognition (e.g., [29,32,33]). Whether or not CI users are in fact worse than hearing controls in this task—as our accuracy and RT data suggest—should be further investigated in larger samples.

Our results furthermore suggest that all CI users, including those with congenital deafness onset, link auditory and visual emotional stimuli, despite their impaired affective prosodic recognition capacities relative to controls and despite their higher reliance on facial expressions. Although all CI users showed significantly larger visual interference than their controls in the Voice task, they did consider the auditory signal in the incongruent condition of the Voice task–that is, they classified the auditory rather than the visual information. In contrast to our hypothesis, this result suggests that the CI users processed the combined crossmodal input, even after congenital deafness. Indeed, the fact that the CD CI users’ vocal emotion recognition was modulated not only by concurrently presented incongruent, but also congruent visual information points to the possibility that multisensory emotion processing abilities can be acquired after a prolonged period of auditory deprivation from birth (note that our CD CI users were implanted between 1 and 42 years of age, at 11.57 years on average). It could be argued that the ability to link crossmodal emotional stimuli depends upon age at cochlear implantation in CD CI users (i.e., upon duration of deafness). However, we did not find a significant correlation between neither the congruency nor the incongruency effect in the Voice task and age at implantation in this group. Importantly, in the Face task we found congruency effects which did not differ between CD CI users and controls. Although a null result cannot be taken as a strong result, the absence of a difference between the CD CI users and their controls in the congruency effect of the Face task goes in the direction of the suggestion that the ability to link audio-visual emotional signals does not depend on early (multi)sensory experience.

It has to be discussed why the LD CI users, other than the CD and ED CI users, displayed no larger congruency effect than their controls for affective prosodic recognition. A possible reason for this finding might be that LD CI users dispose of relatively higher auditory-only affective prosodic recognition skills compared to CD and ED CI users and were thus more similar in auditory performance to the controls. Alternatively, the controls for the LD CI users could have displayed worse auditory-only performance compared to the other controls, rendering possible a larger gain from congruent facial cues. Correlational analyses indeed suggested that in the CI users, a later age at deafness onset as well as a higher age at testing (note that the LD CI users were on average around 20 years older than the CD and ED CI users) was beneficial for auditory-only affective prosodic recognition, whereas in the controls, auditory-only affective prosodic recognition decreased with age. These results indicate that age at deafness onset affects auditory-only affective prosodic recognition accuracy in CI users. It appears that typical auditory experience during (at least) the first three years of life may be beneficial for later affective prosody perception with the CI. Probably, LD CI users are able to match the impoverished affective prosody information coming from the CI with representations learned early in life. In support of this idea is the theory that the first 3–4 years of life form a sensitive period for central auditory system development [22,23]. Although the LD CI users did not reach the performance level of typically hearing individuals (note that the LD CI users had on average about eight years of CI experience), which speaks for a genuine limitation of the CI as a device (as discussed above), they probably profited from their early hearing experience. Second, these results point to decreasing affective prosody recognition capabilities in typically hearing individuals with increasing age. Such an aging effect has been found in earlier studies as well (e.g., [47–49]). From these studies, it is not yet clear how this effect relates to the general age-related decline in acoustic hearing (presbyacusis) that starts at around 40 years of age (e.g., [50–52]). The fact that the CI users in the present study did not show a corresponding aging effect on affective prosodic recognition suggests that deficits in affective prosody perception at least partially result from age-related hearing impairment: Presbyacusis is thought to be caused by damages to the inner ear structures, which are bypassed in electrical hearing with the CI (e.g., [17,23,53]).

In light of this reasoning, the question arises why the LD CI users–just as the CD and ED CI users—experienced a larger interference from incongruent faces on affective prosodic recognition than their controls. A possible account would require that congruency effects and incongruency effects represent distinct processes. Recent evidence suggests that this might be the case. For example, Diaconescu et al. [54] presented healthy participants with visual, auditory, and crossmodal congruent and incongruent stimuli while recording magnetoencephalography (MEG). The stimuli consisted of pictures and sounds of animals, musical instruments, automobiles, and household objects. In one task, participants were supposed to decide whether or not the crossmodal stimuli were semantically related. In this task, the authors found larger responses to incongruent compared to congruent stimulation in regions of the left prefrontal cortex (dorsomedial, centrolateral), suggesting that semantically congruent and incongruent stimuli are processed differently.

Further electrophysiological studies might be helpful to better understand how emotionally congruent and incongruent stimuli are processed by CI users and whether these processes differ as a function of age at deafness onset.

## Conclusions

Our results suggest that CI users are able to link audio-visual emotional stimuli, even after congenital deafness. However, in judging affective prosody they appear overall impaired and more strongly biased by concurrent facial information than typically hearing individuals.

## Supporting information

S1 Text (DOCX)

S2 Text (DOCX)

S1 Fig Accuracy in the voice and the face task. Accuracy rates (in percent) in the congenitally deaf (n = 7), early deaf (n = 7), and late deaf (n = 13) CI users and their respective controls, separately for task (Voice task, Face task) and condition (unimodal, congruent, incongruent). Error bars denote standard deviations. P-values indicate (marginally) significant group differences per condition and condition differences per group in the Voice task as well as a main effect of group in the Face task.
(TIF)

S2 Fig RTs in the voice and the face task. Mean reaction time (RT, ms) of emotion recognition in the congenitally deaf (n = 7), early deaf (n = 7), and late deaf (n = 13) CI users and their respective controls, separately for task (Voice task, Face task) and condition (unimodal, congruent, incongruent). Error bars denote standard deviations. Significant group differences are indicated accordingly.
(TIF)
