# Supplementary material for: Multisensory emotion perception in congenitally, early, and late deaf CI users
Source: PLoS One. 2017 Oct 12;12(10):e0185821. doi: 10.1371/journal.pone.0185821 (PMC5638301; doi:10.1371/journal.pone.0185821)
Supplement: S1 Text — (DOCX) [file pone.0185821.s001.docx]

**S1 Text**

**Results**

**Accuracy rate: Voice task**

*All control participants (n = 26):* The ANOVA showed that the repeated measures factor Condition was significant (*F*(2, 50) = 35.46, *p* < .001). Post hoc comparisons revealed that the controls were significantly more accurate in the congruent condition as compared to the unimodal condition (*t*(25) = 7.17, *p* < .001; congruency effect) and the incongruent condition (*t*(25) = 8.35, *p* < .001). The unimodal and incongruent conditions did not significantly differ (*t*(25) = 1.53, *p* = .14; no incongruency effect).

*CD CI users and controls for CD CI users:* The ANOVA displayed a main effect of Group (*F*(1, 12) = 74.15, *p* < .001), indicating that the CD CI users performed overall less accurately than their matched controls (CD CI users: mean = 54.32 %, SD = 22.71 %; controls: mean = 86.17 %, SD = 8.61 %). There was a significant main effect of Condition as well (*F*(2, 24) = 25.97, *p* < .001) and the Group x Condition interaction was significant (*F*(2, 24) = 9.34, *p* < .01), suggesting that the effect of Condition on accuracy differed between the groups.

Post hoc group comparisons for each condition showed that the CD CI users performed worse than their controls in all three conditions (all t-tests *p* < .05; see S1 Fig). Repeated measures ANOVA on the effect of Condition showed that the conditions significantly differed in the CD CI users (*F*(2, 12) = 22.55, *p* < .001), but only marginally in the controls (*F*(2, 12) = 4.32, *p* = .06). As illustrated in S1 Fig, the CD CI users were significantly more accurate in the congruent relative to both the unimodal (*t*(6) = 3.83, *p* = .01) and the incongruent condition (*t*(6) = 5.19, *p* = .01). Furthermore, they responded significantly more accurately in the unimodal as compared to the incongruent condition (*t*(6) = 4.74, *p* = .01).

**S1 Fig.** **Accuracy in the Voice and the Face Task.** Accuracy rates (in percent) in the congenitally deaf (n = 7), early deaf (n = 7), and late deaf (n = 13) CI users and their respective controls, separately for task (Voice task, Face task) and condition (unimodal, congruent, incongruent). Error bars denote standard deviations. P-values indicate (marginally) significant group differences per condition and condition differences per group in the Voice task as well as a main effect of group in the Face task

Comparisons of the congruency and incongruency effects (i.e., accuracy differences between the congruent/incongruent and the unimodal condition) affirmed group differences concerning the effect of Condition on accuracy: The CD CI users had a significantly larger incongruency effect (*t*(11) = -2.30, *p* = .04) and a marginally significantly larger congruency effect (*t*(7) = 2.15, *p* = .07) as compared to their controls.

*ED CI users and controls for ED CI users:* The ANOVA showed a main effect of Group (*F*(1, 12) = 81.15, *p* < .001), indicating overall lower accuracy in the ED CI users than their controls (ED CI users: mean = 50.90 %, SD = 13.37 %; controls: mean = 85.01 %, SD = 8.99 %). The main effect of Condition was significant as well (*F*(2, 20) = 47.40, *p* < .001), indicating that accuracy differed across conditions. In addition, the Group x Condition interaction was significant (*F*(2, 24) = 4.19, *p* = .03), suggesting that the effect of Condition on accuracy differed between the groups.

In post hoc comparisons, the ED CI users were found to perform less accurate than their controls in all three conditions (all *t*-tests *p* < .05; see S1 Fig). Repeated measures ANOVA showed that the effect of Condition was significant in both the ED CI users (*F*(2, 12) = 33.28, *p* < .001) and their controls (*F*(2, 12) = 14.81, *p* < .001). As illustrated in S1 Fig, the ED CI users performed significantly more accurate in the congruent as compared to both the unimodal condition (*t*(6) = 3.27, *p* = .02) and the incongruent condition (*t*(6) = 10.00, *p* < .001). The unimodal and the incongruent condition significantly differed as well (*t*(6) = 4.30, *p* = .02). The controls were significantly more accurate in the congruent as compared to both the unimodal (*t*(6) = 4.52, *p* = .01) and the incongruent condition (*t*(6) = 5.39, *p* = .01).

Comparisons of the congruency and incongruency effects (i.e., accuracy differences between the congruent/incongruent and the unimodal condition) revealed that the ED CI users displayed a marginally significantly larger incongruency effect as compared to their controls (*t*(11) = -1.76, *p* = .10). The congruency effect did not significantly differ between the CI users and controls (*t*(8) = 0.88, *p* = .40).

*LD CI users and controls for LD CI users:* The ANOVA displayed a main effect of Group (*F*(1, 23) = 32.33, *p* < .001), indicating that the LD CI users had overall lower accuracy than their controls (LD CI users: mean = 5089.85 ms, SD = 2288.70 ms; controls: mean = 2534.91 ms, SD = 721.60 ms). The main effect of Condition was significant as well (*F*(2, 46) = 61.46, *p* < .001), indicating that accuracy differed across conditions. In addition, the Group x Condition interaction was significant (*F*(2, 46) = 11.63, *p* < .001), suggesting that the effect of Condition on accuracy differed between the groups.

In post hoc comparisons, the LD CI users were shown to perform less accurately than their controls in all three conditions (all *t*-tests *p* < .05; see S1 Fig). Repeated measures ANOVA showed that the effect of Condition was significant in both the LD CI users (*F*(2, 24) = 44.79, *p* < .001) and their controls (*F*(2, 22) = 22.01, *p* < .001). As illustrated in S1 Fig, the LD CI users’ performance was significantly more accurate in the congruent as compared to the unimodal condition (*t*(12) = 2.79, *p* = .02). Furthermore, the LD CI users performed significantly more accurately in both the unimodal and the congruent as compared to the incongruent condition, respectively (*t*(12) = 6.72, *p* < .001; *t*(12) = 9.14, *p* < .001). The controls were more accurate in the congruent as compared to the unimodal and the incongruent condition, respectively (*t*(11) = -3.88, *p* < .01; *t*(11) = 7.40, *p* < .001), whereas the unimodal and incongruent conditions did not significantly differ (*t*(11) = 0.76, *p* = .46).

Comparisons of the congruency and incongruency effects (i.e., accuracy differences between the congruent/incongruent and the unimodal condition) demonstrated that LD CI users and controls differed with regard to the effect of Condition on accuracy. The LD CI users displayed a significantly larger incongruency effect as compared to their controls (*t*(22) = -4.55, *p* < .001). The congruency effect did not significantly differ between the LD CI and their controls (*t*(21) = -0.81, *p* = .43).

*Additional analyses:* Additional analyses were computed to test whether the CI users performed at a level different from 0 % correct in the incongruent condition of the Voice task, in which they had particularly low performance. An accuracy level around zero would indicate that the CI users in fact classified the facial expressions instead of the prosody. One-sample *t*-tests showed that all CI groups did perform significantly above 0 % (CD CI users: *t*(6) = 5.47, *p* < .01; ED CI users: *t*(6) = 14.45, *p* < .001; LD CI users: *t*(12) = 10.82, *p* < .001).

Furthermore, we additionally compared the auditory-only prosodic discrimination accuracy between the CD, ED, and LD CI users on the one hand and the controls for the CD, ED, and LD CI users on the other hand. These analyses were run to better understand the group differences found for the congruency effect in the Voice task. Correlational analyses across all CI users and all controls, respectively, indicated that these differences reflected an age effect: Whereas age was significantly positively related to auditory-only prosodic discrimination in the CI users (*t*(25) = 2.13, *p* = .04), it was significantly negatively related to this ability in the controls (*t*(24) = -2.80, *p* = .01).

**Accuracy rate: Face task**

*All control participants (n = 26):* The repeated measures factor Condition was significant (*F*(2, 50) = 10.56, *p* < .001). Post hoc comparisons showed that the controls were significantly more accurate in the congruent condition as compared to the unimodal condition (*t*(25) = 3.01, *p* = .01; significant congruency effect) and the incongruent condition (*t*(25) = 4.01, *p* < .01). The unimodal and incongruent conditions did not significantly differ (*t*(25) = 1.62, *p* = .12; no incongruency effect).

*CD CI users and controls for CD CI users:* The ANOVA showed a significant main effect of Group (*F*(1, 12) = 8.20, *p* = .01), indicating that the CD CI users performed overall worse than their controls (CD CI users: M = 84.93%, SD = 5.82%; controls: M = 91.18%, SD = 7.08%; see S1 Fig). There was a significant main effect of Condition as well (*F*(2, 24) = 3.18, *p* = .06). Post hoc comparisons revealed that the CD CI users and their controls responded more accurate in the congruent as compared to the unimodal condition (*t*(13) = 3.04, *p* = .03). Neither the congruent and incongruent condition (*t*(13) = 1.64, *p* = .25), nor the unimodal and incongruent condition (*t*(13) = -0.61, *p* = .55) significantly differed.

*ED CI users and controls for ED CI users:* The ANOVA did not reveal any significant effect.

*LD CI users and controls for LD CI users:* The ANOVA displayed a main effect of Condition (*F*(2, 46) = 9.54, *p* < .001). Post hoc comparisons showed that both groups responded significantly more accurately in the congruent as compared to both the unimodal (*t*(24) = -2.97, *p* = .01) and the incongruent condition (*t*(24) = 3.73, *p* < .01). The unimodal and incongruent conditions did not significantly differ (*t*(24) = 1.10, *p* = .28).
