# Supplementary material for: Multisensory emotion perception in congenitally, early, and late deaf CI users
Source: PLoS One. 2017 Oct 12;12(10):e0185821. doi: 10.1371/journal.pone.0185821 (PMC5638301; doi:10.1371/journal.pone.0185821)
Supplement: S2 Text — (DOCX) [file pone.0185821.s002.docx]

**S2 Text**

**Results**

**Reaction time: Voice task**

*All control participants (n = 26):* The ANOVA showed that the repeated-measures factor Condition was not significant (*F*(2, 50) = 1.40, *p* = .26).

*CD CI users and controls for CD CI users:* The ANOVA did not reveal any significant effect.

*ED CI users and controls for ED CI users:* The ANOVA displayed a significant main effect of Group (*F*(1, 12) = 5.09, *p* = .04), indicating that the ED CI users responded overall significantly slower than their controls (ED CI users: M = 2364.00 ms, SD = 401.49 ms; ED CI controls: M = 1950.00 ms, SD = 279.65 ms; see S2 Fig).

**S2 Fig.** **RTs in the Voice and the Face Task.** Mean reaction time (RT, ms) of emotion discrimination in the congenitally deaf (n = 7), early deaf (n = 7), and late deaf (n = 13) CI users and their respective controls, separately for task (Voice task, Face task) and condition (unimodal, congruent, incongruent). Error bars denote standard deviations. Significant group differences are indicated accordingly.

*LD CI users and controls for LD CI users:* The ANOVA displayed a significant main effect of Group (*F*(1, 23) = 15.57, *p* < .001), indicating that the LD CI users responded overall significantly slower than their controls (LD CI users: M = 2754.95 ms, SD = 659.98 ms; LD CI controls: M = 1935.44 ms, SD = 370.98 ms; see S2 Fig).

**Reaction time: Face task**

*All control participants (n = 26):* The ANOVA showed that the repeated-measures factor Condition was not significant (*F*(2, 50) = 1.72, *p* = .98).

*CD CI users and controls for CD CI users:* The ANOVA did not reveal any significant effect.

*ED CI users and controls for ED CI users:* The ANOVA did not reveal any significant effect.

*LD CI users and controls for LD CI users:* The ANOVA showed a significant main effect of Group (*F*(1, 23) = 5.59, *p* = .03), indicating that the LD CI users responded overall significantly slower than their controls (LD CI users: M = 2238.80 ms, SD = 401.84 ms; LD CI controls: M = 1919.22 ms, SD = 300.19 ms; see S2 Fig).
