# Supplementary figures and images for: Multisensory emotion perception in congenitally, early, and late deaf CI users
Source: PLoS One. 2017 Oct 12;12(10):e0185821. doi: 10.1371/journal.pone.0185821 (PMC5638301; doi:10.1371/journal.pone.0185821)

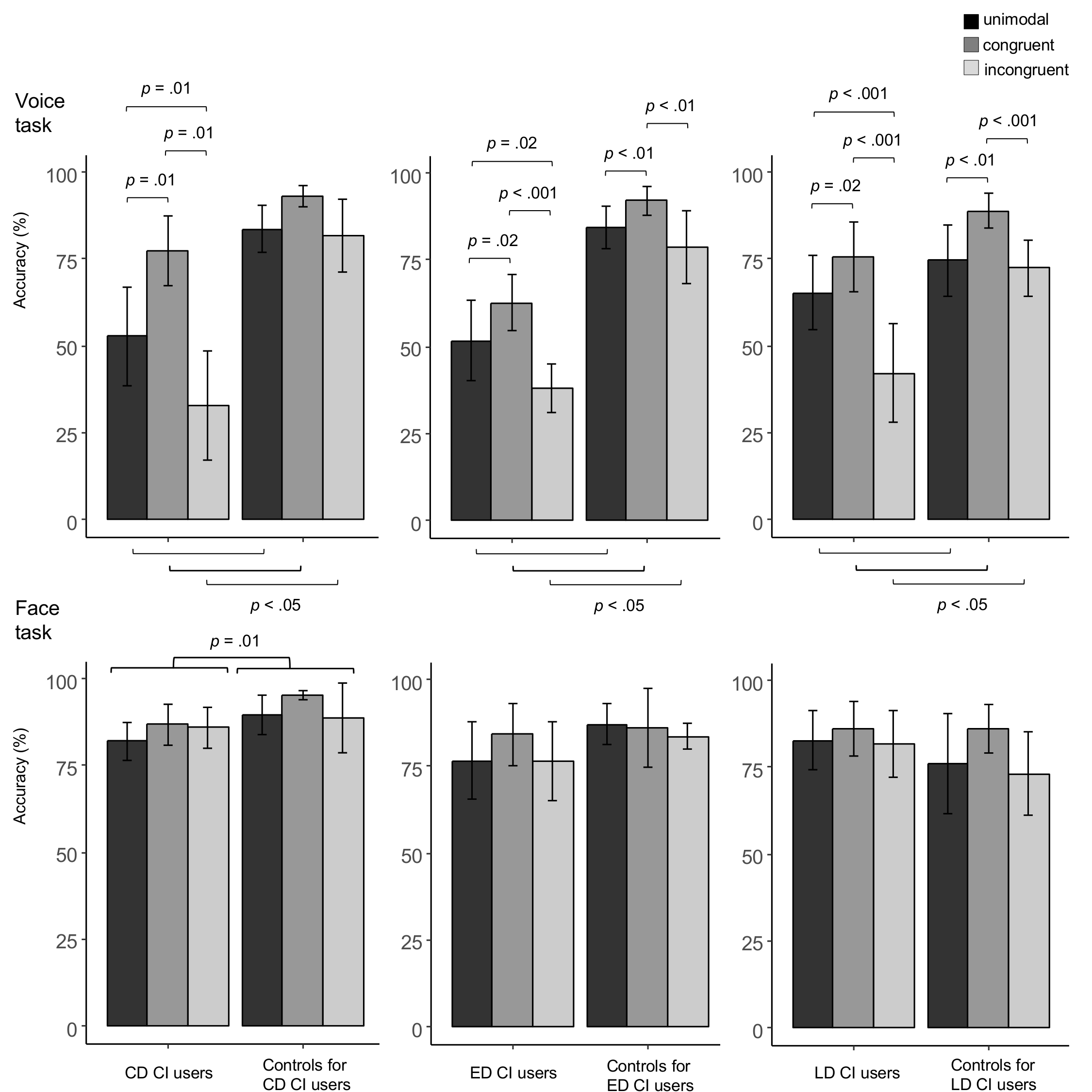

Supplement: S1 Fig — Accuracy rates (in percent) in the congenitally deaf (n = 7), early deaf (n = 7), and late deaf (n = 13) CI users and their respective controls, separately for task (Voice task, Face task) and condition (unimodal, congruent, incongruent). Error bars denote standard deviations. P-values indicate (marginally) significant group differences per condition and condition differences per group in the Voice task as well as a main effect of group in the Face task. (TIF) [file pone.0185821.s003.tif]

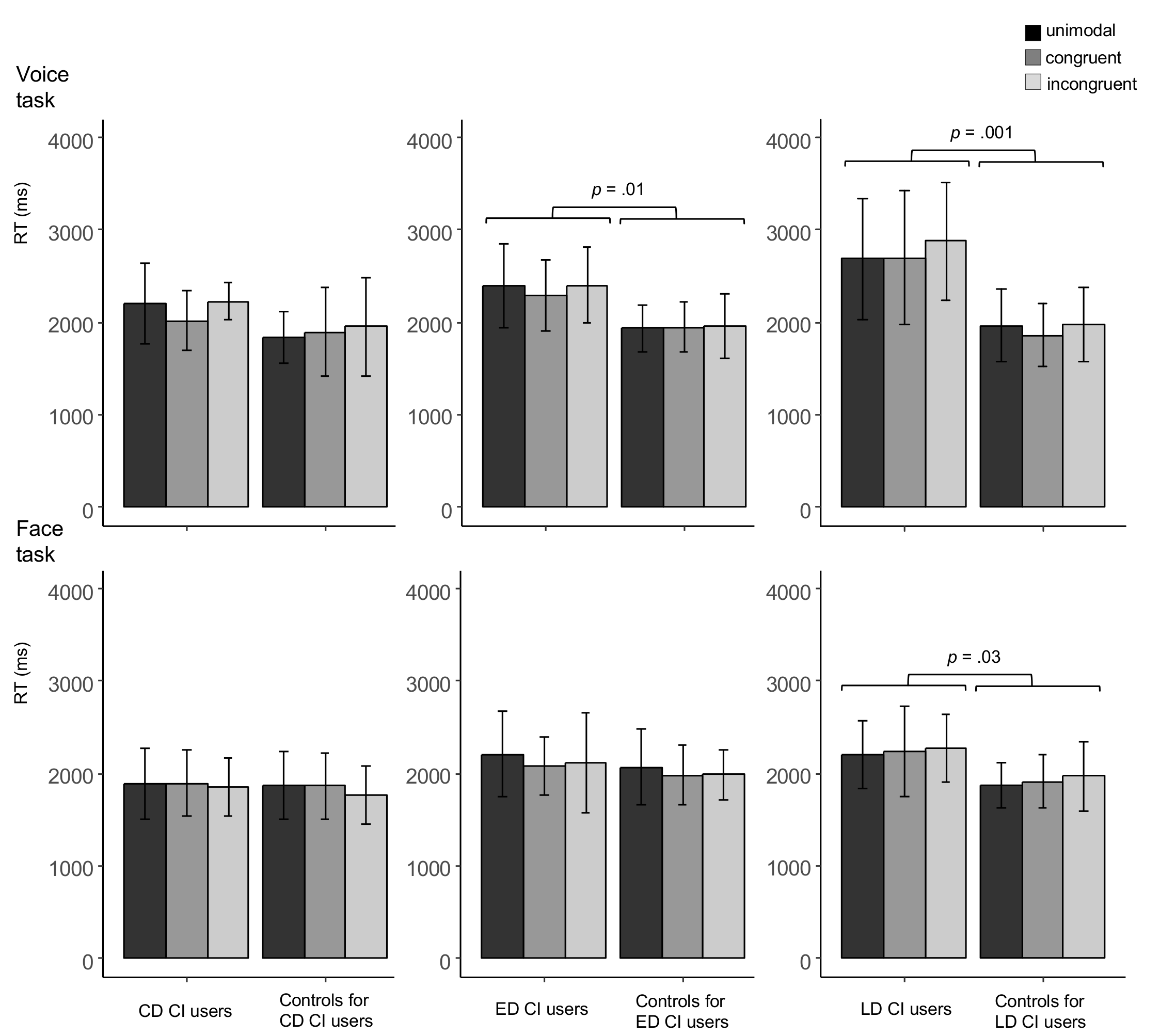

Supplement: S2 Fig — Mean reaction time (RT, ms) of emotion recognition in the congenitally deaf (n = 7), early deaf (n = 7), and late deaf (n = 13) CI users and their respective controls, separately for task (Voice task, Face task) and condition (unimodal, congruent, incongruent). Error bars denote standard deviations. Significant group differences are indicated accordingly. (TIF) [file pone.0185821.s004.tif]
